# Effects of an open-label placebo intervention on reactions to social exclusion in healthy adults: a randomized controlled trial

**DOI:** 10.1038/s41598-023-42547-7

**Published:** 2023-09-16

**Authors:** Leonie Stumpp, Melissa Jauch, Dilan Sezer, Jens Gaab, Rainer Greifeneder

**Affiliations:** 1 Resilience Centre Switzerland, Basel, Switzerland; 2 Division of Social Psychology, Faculty of Psychology, University of Basel, Basel, Switzerland; 3 Division of Clinical Psychology and Psychotherapy, Faculty of Psychology, University of Basel, Basel, Switzerland

**Keywords:** Human behaviour, Psychiatric disorders

## Abstract

Social exclusion, that is being left out by others, can have adverse consequences for individuals’ psychological well-being. Even short-term experiences of social exclusion strongly threaten basic psychological needs and cause so-called *social pain.* Prior research suggests an overlap between the experience of social and physical pain that, amongst others, is reflected by the effectiveness of physical pain treatments in alleviating social pain. Drawing upon these prior findings, we here explore whether open-label placebos, which have previously been found to be effective in reducing physical pain, can alleviate social pain following social exclusion. Seventy-four healthy participants were randomly assigned to one of four conditions in a 2 × 2 between-subjects design: First, they either received an open-label placebo intervention or no treatment. Second, they either experienced inclusion or exclusion by their co-players in the interactive ball-tossing game Cyberball. We find that excluded participants in the open-label placebo condition experienced significantly less hurt feelings compared to those in the control condition (Cohen’s *d* = 0.77). There was no effect of treatment for need threat. The findings suggest new possibilities to alleviate social pain, which is of particular interest in the context of preventing destructive and maladaptive behaviors in situations where functional coping strategies are unavailable.

## Introduction

*Social exclusion* by other people can have severe negative effects for individuals’ well-being because it threatens fundamental psychological needs such as the need for belonging or control^1^. Research further suggests that frequent experiences of social exclusion are associated with an increased risk for the development of mental disorders such as depression^2,3^, anxiety disorders^4^, and personality disorders (e.g., borderline personality disorder^5,6^).

From a conceptual perspective, it is often argued that the immediate experiences provoked by social exclusion reflect *social pain*, which resembles the experience of physical pain in several regards. *Pain-overlap theory*, for instance, holds that both social and physical pain rely on similar neural pathways and serve as warning signals to inform individuals that their well-being is endangered^7–11^. Consistent with this overlap of physical and social pain, some studies show that treatments applied to mitigate physical pain including both active medication (e.g., acetaminophen or marijuana^12,13^) and placebo treatment^14^ can be effective in mitigating the experience of social pain, too.

Building upon the notion of a physical-social pain overlap, the present work tests a new intervention in the context of social pain by examining the effect of an *open-label placebo* intervention on the experience of social pain following social exclusion. In contrast to deceptive placebos, open-label placebos are administered with full disclosure about the fact that a substance does not contain active medication and have been shown to be effective in improving symptoms across several conditions including chronic and acute pain^15,16^.

The temporal need-threat model of ostracism or social exclusion holds that reactions to exclusion can be differentiated in three temporal stages: (1) a reflexive stage which is characterized by strong, negative reactions in immediate response to the exclusion event; (2) a reflective stage which is characterized by the onset of coping strategies as soon as reflexive responses decrease; and (3) a resignation stage which occurs when recovery from the exclusion event was not successful^17^.

The reflexive stage that occurs immediately after exclusion cues have been detected is characterized by the automatic reaction of the so-called ostracism detection system. This alarm system is presumed to inform individuals that their status of inclusion is endangered by eliciting feelings of social pain as well as a threat to the four fundamental needs for belonging, self-esteem, control, and meaningful existence^3,17^. From an evolutionary perspective, this high sensitivity to exclusion was useful, as in earlier times exclusion by one’s social group was potentially life-threatening and the timely detection of exclusion cues was crucial for one’s survival^11^. As a result of this alarm function of social pain, situational factors play a minor role for the experience of pain. For instance, just as individuals experience physical pain after touching a flame regardless of the source of the flame, social pain resulting from social exclusion hurts regardless of the exclusion source^18^. Consistent with the theoretical notion of a robust alarm function, the reflexive social pain reaction has been shown to be relatively insensitive to moderation. Previous research could only identify few strategies to buffer social pain and need threat directly alter the exclusion experience (for a review, see^19^, but see also^20^ for a different perspective). However, the similarity between social and physical pain is not limited to their function as an alarm system, but also extends to underlying mechanisms, e.g., the pain pathway.

The pain pathway starts in nociceptors in the skin, muscle, or internal organs, which generate pain signals as a reaction to a physical, chemical, or inflammatory stimulus. These pain signals are integrated in the spinal cord, where some of them cause an immediate defensive response, for example muscle contraction to leave the pain-causing condition. The other signals are continuing their way up to the thalamus, where they are transmitted to the pain-related regions in the higher-level cortex such as the primary somatosensory cortex (S1), the dorsal anterior cingular cortex (dACC), the dorsolateral prefrontal cortex (DLPFC) and the anterior and posterior insula^21^. Pain-overlap theory holds that these underlying neural as well as psychological mechanisms are similar for physical and social pain^7–11^. Just as experiencing physical pain signals individuals that their physical well-being is endangered, social pain may signal individuals that their social well-being (i.e., inclusion into a group) is endangered^11^. Several functional magnetic resonance imaging (fMRI) studies support this theory, showing that social exclusion activates similar brain areas as physical pain. In particular, this concerns the brain areas which are related to the emotional components of physical pain as the dACC, the anterior insula and two regions of the right ventrolateral prefrontal cortex (rVLPFC)^8,21–23^. Activity of the dACC was found to be associated with self-reported distress while activity of the anterior insula did not correlate with self-reported distress. Activity of the rVLPFC was again found to be negatively correlated with self-reported distress and ACC activity during social exclusion^8^. Findings of transcranial magnetic stimulation studies highlight the role of the rVLPFC in the emotion regulation of social pain^23,24^. Interestingly, the activation of the rVLPFC only resulted in successful emotion regulation when experiencing social pain but not when experiencing physical pain^24^. These findings may support the connectivity of dACC and rVLPFC, in a way that the rVLPFC takes a self-regulating role in weakening the distressing effects of social exclusion, which again are related to the activity of the dACC^8,24^. The latter is often put into focus when it comes to social exclusion, because it plays an important role in the detection of social exclusion due to its assumed role as a neural alarm system^10,25^. The dACC thus seems to play a key role in experiences of social exclusion as well as feelings of distress and depression, as for example hypoactivity in these dorsal areas has been observed in depressed patients^26,27^.

Besides the neuronal overlap between experiencing social and physical pain, there is an overlap in psychological responses to both types of pain. For instance, participants who are asked to recall a past episode of physical pain and their psychological experiences report lower self-esteem, poorer control, increased negative affect, and desire to aggress. Importantly, these psychological experiences of physical pain were very similar to the experiences participants report when asked to recall an episode of social pain^28^. Consistent with these findings, a short induction of physical pain (i.e., holding one’s hand in cold water) threatened participants’ psychological needs and affect in a similar way as an induction of social pain (i.e., being excluded in an online-ball tossing game)^28^.

Further consistent with the evidence suggesting common psychological and neural mechanisms underlying social and physical pain^9,22^, research suggests that treatments applied to mitigate the experience of physical pain, such as the application of pain killers, are also effective in mitigating social pain. For instance, two experiments by DeWall et al.^13^ provide evidence for the efficacy of acetaminophen (paracetamol outside of the US) in reducing social pain. In their first study, a daily dose of the painkiller over a period of three weeks reduced the frequency of self-reported hurt feelings. The second study including fMRI suggests that acetaminophen compared to placebo intake significantly inhibits dACC activation when being excluded in the Cyberball game, even though participants in the drug condition do not report less social distress after being excluded than participants in the placebo condition.

Importantly, the efficacy of pain treatments in the context of social pain is not restricted to active medication, but also extends to placebo treatment. Previous research has shown that a placebo nasal spray containing only saline solution reduces rejection-related negative affect. Additionally, fMRI indicates that the placebo treatment for rejection-related pain increases activity in the same prefrontal-brainstem pathways as a placebo treatment for physical heat pain^14^.

Placebo treatments are widely used in clinical practice^29^ as well as in research. Placebos are defined as therapeutic procedures that have, based on the underlying therapeutic theory, no intended effects on the condition treated^30,31^. The common conception of placebos involves deception of the patients which causes an ethical problem due to violation of the person’s autonomy^32^. To bypass this ethical dilemma, an increasing amount of research examined the efficacy and mechanisms of so-called open-label placebos (OLPs)^18^. Unlike participants receiving deceptive placebos, participants receiving OLP are explicitly informed that they receive a placebo treatment, e.g., a pill without an active ingredient. In contrast to earlier theoretical accounts arguing that deception is as a necessary component for the efficacy of placebos^33^, there is now support for the effectiveness of OLP in several domains^16,32^. For instance, OLP pills can improve symptoms of irritable bowel syndrome^34^, allergic rhinitis^35^, cancer-related fatigue^36^, chronic back pain and related disabilities^37^, and migraine attacks^38^.

Current research tries to explain why OLPs might be effective. While the mechanisms of deceptive placebos are relatively well studied, the underlying processes of OLP effects yet remain largely unclear. This is mainly because deception and the corresponding expectancies of receiving an active drug, which were long believed to be the driving components of placebo treatments, are not involved in OLP^39^. Few studies support the different working mechanisms of deceptive placebos and OLPs. For example, dispositional optimism relates to deceptive placebo effects but not to OLP effects^40^ and treatment expectancies are believed to play a less important role in OLP than in deceptive placebo treatments^41^. In terms of the specific OLP effects, research proposes three mechanisms: (1) pharmacological memory, (2) the influence of a treatment rationale, and (3) “embodied” consciousness^32,39,42^. The (1) pharmacological memory explanations states that taking any pill, even when explicitly labelled as placebo, triggers associations with taking an active drug, thus eliciting conditioned responses. These conditioned responses are similar to physiological responses to active drugs^32^. The second explanation (2) highlights the influence of a treatment rationale provided by the experimenter to the individual containing the potential effects of OLPs in a friendly, trustworthy, and empathetic manner^42,43^. Research suggests that a comprehensible rationale for why OLPs work intensifies the effects of OLPs, providing support for the potential mechanism of conscious, positive expectations^42^. However, previous research could identify neither belief in the power of OLPs nor expectations as a mechanism of OLP effects^44^. Nevertheless, state-of-the-art OLP procedures hold that the rationale should be communicated to the individual by the experimenter prior to the intake. Consistent with this procedural set-up, positive framing and instilling hope for improvement can modulate the central regulation of pain^32^. The third explanation (3), embodied cognition, holds that humans’ physical interaction with the world has an impact on cognitions^45^. According to this concept, OLPs lead to specific cognitions by stimulating bodily sensations. This can be explained by the underlying assumption that motor behaviour shapes cognition^46^. For instance, the motor action of swallowing the OLP-pill may shape cognitions regarding pain perception, resulting in the production of pain-relieving endogenous substances in the brain. This approach emphasizes the role of the bodily sensations in the formation of conditioned responses and refers to the idea that the body can react to a stimulus automatically^32,39,46^. Importantly, the three above mentioned mechanisms are not mutually exclusive and most likely, OLP effects depend on all these three mechanisms and their interaction^32^.

Importantly, OLPs have not only been shown to mitigate symptoms of physical diseases such as pain^37,47^, but research also suggests positive effects on symptoms of psychiatric disorders such as depression^48^, general psychological well-being^49^, and the self-conscious emotion of guilt^50^. Intriguingly, some research suggests that placebos seem to primarily reduce self-reported symptoms (e.g., side effects of cancer treatment as fatigue, nausea, and pain) instead of objectively measurable symptoms (e.g., the size of a malignant tumor)^51^. This is also supported by a study that examined the effect of OLPs on experimentally induced sadness. While the OLP compared to the control condition reported less self-reported sadness, physiological parameters, such as heart rate, did not differ as a function of treatment condition^52^. Yet, although prior OLP research has mainly focused on symptoms by self-report (e.g. Nurko et al.^53^) others also have shown that OLPs have effects on objective measures (i.e. exam grades^54^, neuronal markers for emotional distress^44^, reduced cortisol^55^) and that OLP effects can be clocked by the opioid antagonist Naloxon^56^. OLPs thus may be able to alter both self-reported as well as objectively measurable physical and psychiatric symptoms.

However, so far, the effect of an OLP on social pain has not yet been examined.

As discussed before, previous research suggests that social and physical pain share parts of the same underlying neuronal pain-processing-system with particularly the dACC playing an important role for physical as well as social pain detection^7–11^. As a consequence, established treatment methods for physical pain may also be applied to relieve social pain^13^. OLP treatment is such a method, which however has not yet been investigated in the context of social pain. The present research aims at closing this gap and at contributing new evidence regarding the social and physical pain overlap. Moreover, evidence for the effectiveness of OLPs in mitigating social pain has important practical implications in the context of mental disorders, particularly for individuals not able to rely on functional coping strategies^57^. More specifically, while healthy individuals with functional coping strategies might rely on other strategies to cope with social exclusion, such as cognitive reattributions or seeking social support, certain individuals might lack such strategies. OLP could thus be a promising approach to reduce the immediate distress induced by social exclusion and prevent dysfunctional coping strategies (e.g., aggressive behaviors). Based on the theoretical background, the following hypotheses were tested: (1) excluded participants will report more need threat compared to included participants, (2) excluded participants will report more hurt feelings compared to included participants, (3) excluded participants who receive an OLP pill will experience less need threat compared to excluded participants in a no-treatment condition, and (4) excluded participants who receive an OLP pill will experience less hurt feelings compared to excluded participants in a no-treatment condition.

## Methods

### Study design

We conducted a 2 (treatment: OLP versus no-treatment) × 2 (social experience: inclusion versus exclusion) between-subjects design. Participants were randomly assigned to one of the four conditions. Written informed consent was obtained from each subject before participation in the study. The research was approved by the Ethics Committee of the Faculty of Psychology at the University of Basel, Switzerland, and carried out in accordance with the Declaration of Helsinki. Prior to data collection, the study was pre-registered here: https://aspredicted.org/t3nf9.pdf. In accordance with the journal’s policy and in addition to the a-priori pre-registration, the study was retrospectively registered as a clinical trial on the German Clinical Trials Register (DRKS00031399; 01/03/2023).

### Participants

A power analysis using G*Power^58^ revealed that 128 participants are needed to detect a medium effect size *f* = 0.25 (η^2^ = 0.06) with a power of at least 0.80 and with an alpha error of 0.05 in a 2 × 2 ANOVA design. We thus planned to strive for a sample size of *N* = 128, but pre-registered that data collection might be stopped earlier due to feasibility issues before the full sample size is reached. The assumption of a medium effect size is based on studies using a similar approach, such as a study by Kelley et al.^59^ who observed effect sizes of *d* = 0.54 between OLP and no-treatment for major depression symptoms assessed with the clinician-rated 17-item Hamilton Scale for Depression (HAM-D-17) as dependent variable, and a study by Hahn et al.^52^ who observed effects of *d* = 0.79 between OLP and no-treatment for sadness.

In total 77 participants took part in the study. Participants were mainly university students, but also other individuals affiliated with the university (i.e., employees, former students). Participants either received course credit (psychology students) or a monetary compensation. Consistent with pre-registration, a participant’s data was excluded from the analyses if they had indicated low levels of seriousness (*n* = 1) or if they had indicated difficulties in understanding parts of the study (*n* = 1). Moreover, one person was excluded due to technical problems. Thus, 74 observations (51 females, *M*_*age*_ = 27.27, *SD* = 11.64, range = 18 to 60 years) have been considered for the final analyses. Participants were randomly assigned to one out of four conditions: OLP + inclusion (n = 17), OLP + exclusion (n = 21), no-treatment + inclusion (n = 21), and no-treatment + exclusion (n = 15).

Due to time constraints and data collection being complicated by COVID-19 restrictions, the desired sample size was not achieved. Therefore, we conducted a sensitivity analysis based on the achieved sample size. This analysis revealed that with a sample size of *N* = 74, effects of *f* = 0.33 (η^2^ = 0.10) can be found with a power of 0.80 at alpha = 0.05.

### Procedures and materials

The study took place in different rooms on the campus of the University of Basel, Switzerland. First, participants were asked to read and sign the informed consent. The experimenter then informed all participants about the study’s purpose and the way placebo treatments are thought to work. Then, the experimenter randomly assigned participants to the OLP group or the no-treatment group by drawing lots. In the OLP condition, participants received an OLP intervention. This included a rationale (please see Supplemental Material) presented by the experimenter in their own words, focusing on three important discussion points put forward by Kaptchuk et al.^34^: (1) previous research has found a powerful placebo effect, (2) the body can react automatically to taking a pill due to learning mechanisms, and (3) doubts about the efficacy are okay, but a positive expectancy helps. Participants in the no-treatment condition did not receive an intervention but were told that the no-treatment condition is as important as the OLP condition. Both groups were then informed that they were going to play the online ball-tossing game Cyberball during which they might be socially excluded by their co-players, potentially resulting in feelings of social pain. Consistent with the conceptual notion of OLP and prior evidence that providing the rationale is important (e.g.^42^) participants in the OLP condition were told that OLPs are expected to reduce negative affect and feelings of social pain after social exclusion experienced in Cyberball. Then, these participants took one placebo pill. All participants were asked and to sit and wait for two to three minutes, then they were guided to a computer and started the second part of the study.

Participants started by indicating the level of social pain they expected in case of social exclusion (a detailed description and results of this variable is reported in the Supplemental Material) and then were randomly assigned to either an inclusion or exclusion condition in the online ball-tossing game Cyberball^60^. While in the inclusion condition of Cyberball, participants received a similar share of throws as the other players, participants in the exclusion condition only received the ball twice at the beginning of the game and were then ignored by their co-players. In both conditions the game took approximately 2 min and consisted of 30 ball tosses in sum. To check the exclusion manipulation’s success, participants were asked to indicate the extent to which they actively participated in the ball throwing (*1* = *not at all; 9* = *very actively*) and the percentage of throws they had received in the Cyberball game.

Then all participants were asked to indicate their levels of need threat during the game, which was assessed by means of a 4-item need threat scale^20^ (α = 0.97,* M* = 3.82, *SD* = 2.69). This scale focuses on subjective exclusion experiences assessed by four bipolar items presented on a 9-point semantic differential scale. The items cover the need for belonging (“rejected–accepted”), self-esteem (“devalued–appreciated”), control (“powerless–influential”), and meaningful existence (“invisible–noticed”). Higher ratings indicate stronger need threat.

Additionally, the participants were asked to indicate to what extent they currently feel hurt by the other players with a single 9-point Likert-type item (“*The other players’ behavior hurt me*”; 1 = *not at all*, 9 = *very much*; *M* = 3.20, *SD* = 2.47). In addition, general social pain was exploratorily assessed on a 11-point numeric rating scale, which is often used in the context of assessing physical pain in clinical settings (0 = *no pain*, 10 = *strongest pain imaginable*; *M* = 2.51, *SD* = 1.89). Afterwards, several control variables were assessed (see Supplemental Material). Finally, all participants were asked to provide details on their demographics and their participation in the study (e.g., how seriously they had answered all questions).

## Results

RStudio 1.4.1106 was used to compute all statistical analyses. Inferences about statistical significance are based on alpha = 0.05. All means and standard deviations are reported in Table 1. Effect sizes are indicated in partial η^2^ and Cohen’s *d* (for pairwise comparisons).

**Table 1:** Means and standard deviations (in parentheses) as a function of social experience (inclusion vs. exclusion) and placebo condition (OLP vs. NT).

	Participation	Perceived throws	Need threat	Hurt feelings	Social pain
Group					
Exclusion + OLP	2.29 (0.72)	7.67 (5.17)	6.14 (0.80)	4.57 (1.60)	3.43 (1.63)
Exclusion + NT	2.27 (1.10)	6.40 (3.46)	6.52 (1.58)	6.13 (2.39)	5.53 (1.96)
Inclusion + OLP	7.82 (0.73)	34.59 (14.54)	1.71 (1.12)	1.35 (0.49)	1.24 (0.56)
Inclusion + NT	7.76 (1.18)	45.10 (15.53)	1.29 (1.13)	1.24 (0.44)	1.19 (0.51)

Participation, need threat, and hurt feelings were assessed on a scale from 1 to 9. Social pain was assessed on a scale from 0 to 10. Perceived throws are indicated in percent.

*OLP* open-label placebo, *NT* no-treatment.

### Manipulation checks

A two-sided t-test showed a significant effect of social experience on perceived participation, *t*(71.73) = 25.31, *p* < 0.001, *d* = 5.88, indicating that excluded compared to included participants perceived themselves to participate less. Similarly, a two-sided *t*-test yielded a significant effect on perception of received throws, *t*(43.33) = 12.44, *p* < 0.001, *d* = 2.86, in that excluded compared to included participants reported fewer throws.

### Dependent variables

**Figure 1:**
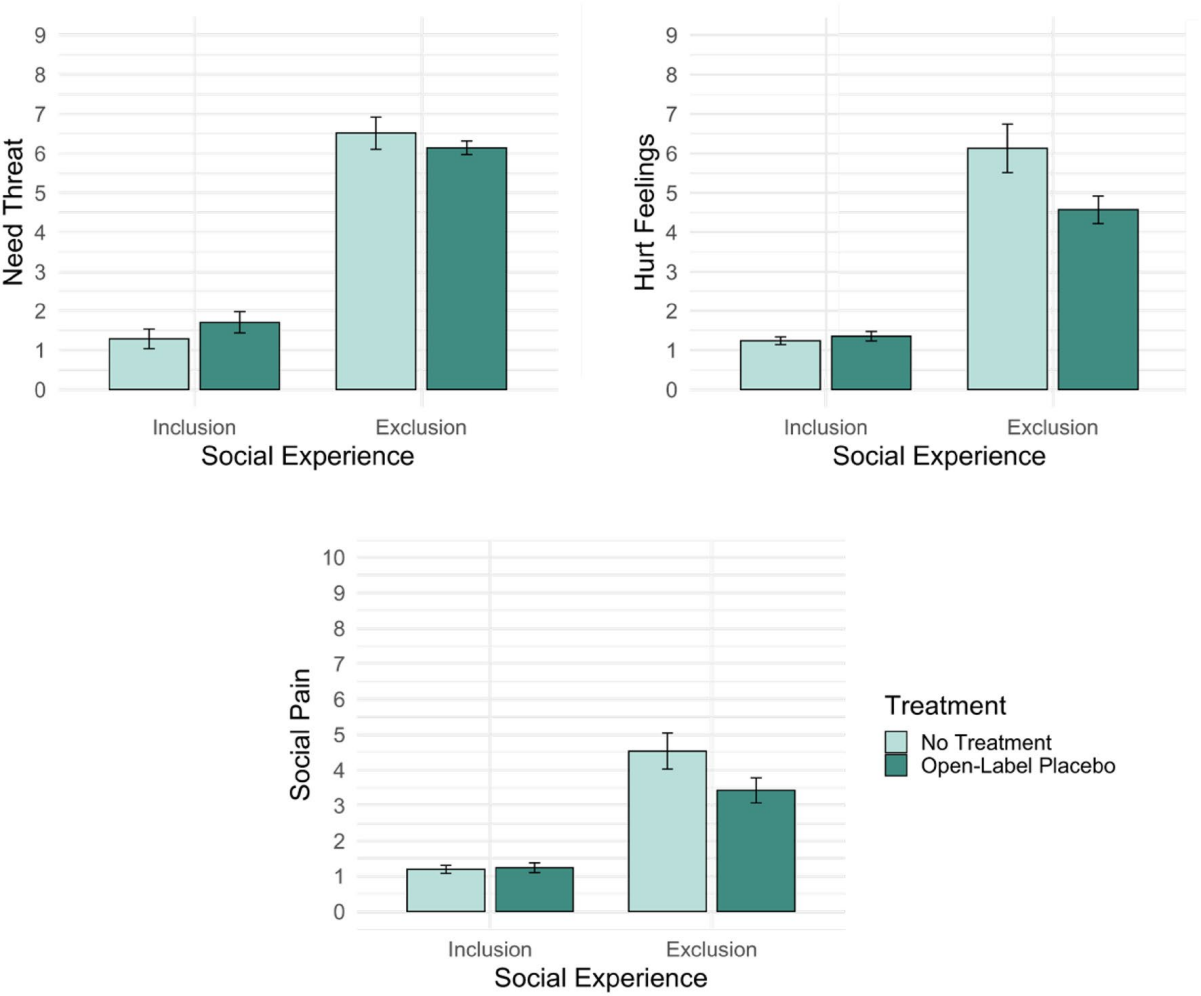
Participants’ levels of need threat, hurt feelings and social pain as a function of treatment and social experience. Error bars represent the standard errors of the mean, *N* = 74.

We pre-registered our hypotheses with regard to hurt feelings and need threat, using scales that have been previously established in social exclusion research^20^. In line with the assessment of physical pain in clinical settings, we additionally assessed an exploratory measure of social pain. The three measures of need threat, hurt feelings and social pain were highly intercorrelated (*r* ranging between 0.75 and 0.88, *p* < 0.001). A 2 × 2 between subjects ANOVA type III was then conducted, separately for the dependent variables need threat, hurt feelings, and the exploratory social pain measure (see Table 2 for coefficients). Need threat, hurt feelings and social pain are not normally distributed and variances among groups are not equal. Still, ANOVAs were conducted, because research showed, that *F*-tests are robust in terms of Type I errors, even if the requirements are not met^61^. Figure 1 depicts participants’ levels of need threat, hurt feelings and social pain as a function of treatment and social experience.

**Table 2:** ANOVA results for the dependent variables need threat, and hurt feelings.

Dependent variable	Independent variable	*F*(1, 70)	*p*	η^2^_p_	95% CI
Need threat	Social experience	318.49***	< 0.001	0.82	[0.73, 0.86]
	Treatment	0.01	0.932	0.00	[0.00, 0.03]
	Social experience × treatment	2.15	0.147	0.03	[0.00, 0.14]
Hurt feelings	Social experience	150.67***	< 0.001	0.68	[0.55, 0.75]
	Treatment	4.79*	0.03	0.06	[0.00, 0.19]
	Social experience × treatment	6.43*	0.013	0.08	[0.00, 0.22]
Social pain	Social experience	82.95***	< 0.001	0.54	[0.37, 0.64]
	Treatment	3.04	0.086	0.04	[0.00, 0.16]
	Social experience × treatment	3.58^†^	0.063	0.05	[0.00, 0.17]

*CI* confidence interval.

^†^*p* < 0.10, *p < 0.05, **p < 0.01, ***p < 0.001.

### Need threat

Analyses revealed a significant main effect of social experience on need threat (*p* < 0.001), as excluded compared to included participants experienced more need threat, thus supporting Hypothesis 1. However, the interaction between social experience and treatment was not significant (*p* = 0.147). Contradicting Hypothesis 3, there was no significant effect of treatment in the exclusion conditions, as excluded participants who received an OLP (compared to no-treatment participants) did not experience less need threat, *F*(1,70) = 0.92, *p* = 0.341, η^2^_p_ = 0.01, *d* = 0.20.

### Hurt feelings

Again, supporting Hypothesis 2, a significant main effect of social experience on hurt feelings was observed (*p* < 0.001), as excluded (compared to included participants) indicated higher levels of the hurt feelings. We also observed a significant main effect of treatment on hurt feelings (*p* = 0.003). Both main effects were qualified by a significant interaction between treatment and social experience (*p* = 0.013). To test Hypothesis 4, we decomposed the significant interaction and calculated simple main effects, which revealed a significant effect of treatment on hurt feelings in the exclusion condition, *F*(1,70) = 10.80, *p* = 0.002, η^2^_p_ = 0.13 (*d* = 0.77), but not in the inclusion condition *F*(1,70) = 0.06, *p* = 0.803, η^2^_p_ < 0.01, (*d* =  − 0.25). Consistent with Hypothesis 4, we thus observe that excluded participants who received an OLP compared to excluded participants in the no-treatment condition experienced lower levels of hurt feelings.

## Discussion

Pain-overlap theory^9^ holds that social pain, for instance elicited by instances of social exclusion, and physical pain share a common underlying neuronal basis. As an intriguing consequence, one may argue that social pain may be treated similarly to physical pain. Consistent with this conjecture, the aim of the present study was to investigate whether an OLP treatment mitigates the adverse psychological consequences of social exclusion, such as experiences of need threat and hurt feelings immediately after being socially excluded. Extending prior research on the effectiveness of OLPs in the context of psychological outcomes^43,48,52,62^ and consistent with pain-overlap theory, results suggest that an OLP pill can buffer feelings of social pain in excluded participants. Contrary to the pre-registered hypotheses, results were not significant for experienced need threat, which may be accounted for by various explanations.

First, pain-overlap theory refers to pain and not need threat, such that conceptual derivations are proximal for hurt feelings and social pain but require a bit of a stretch for need threat. Second, while need threat often entails an objective referent (e.g., not being part), hurt feelings are fundamentally subjective and therefore more malleable. This is in line with prior research showing that expecting exclusion^63^ or making the same exclusion experience repeatedly^64^ does not diminish need threat following social exclusion, speaking to a certain robustness of need threat.

Finally, while the exact mechanisms of OLP effects are not clear yet, some authors suggest that the rationale could be an important component to induce effects^42,50,65,66^. Importantly, while our rationale focused on getting hurt, the threatening character of being excluded for the four fundamental psychological needs was not mentioned at all. Future research should explore whether not only self-reported social pain, but also need threat, can be buffered following a specifically tailored OLP intervention. Consistent with this speculation, the OLP manipulation did not significantly affect feelings in the inclusion group, presumably because the rationale focused on the possible consequences of exclusion only, but not (positive) expectations for being included.

Several limitations to the study deserve mentioning. First, due to feasibility reasons, the aspired sample size of 128 was not achieved. The final sample consisted of 74 participants. With this sample size, only effects bigger than or equal to η^2^ = 0.10 can be detected with a power of 0.80. It is thus conceivable that non-significant effects, such as need threat, were too small to be detected.

Second, we cannot rule out the possibility that differences in the duration of the intervention in the OLP compared to the control group and thus differences in attention confounded the effect. In fact, the interaction between experimenter and participant in the OLP group (about 10 min) was 2 min longer than the interaction in the control group (about 8 min). Importantly, Gaab et al.^43^ showed that a key component of effective placebo treatment is to convey the rationale in a trustworthy, friendly, and empathetic way. Meeting all participants regardless of condition and the actual time taken in the same friendly, trustworthy, and empathetic manner was therefore a priority in the present study to reduce the risk of confounding effects. Moreover, as socially excluded participants are highly susceptible to information, we decided against the possibility of extending the duration of the control group intervention by providing additional, unrelated information. In addition, the control group was designed to control for nonspecific factors of the intervention. As many OLP studies emphasize the central importance of the rationale^42,65,66^, the characteristic components of our OLP treatment were defined to be the pill intake as well as the treatment rationale. Therefore, providing the rationale to the control group was omitted. Thus, while we cannot rule out that differences in the duration between interventions confounded the effect, we can rule out that the effect is driven by any information only participants in the control group received.

Third, we cannot draw any conclusions regarding the durability of the effects found, because within the study design, only a single OLP intake was administered and assessed for its immediate effects. However, given that the immediate reactions to social exclusion are usually experienced most intensely and are assumed to be difficult to buffer by, for instance, cognitive coping strategies^17^, learning how immediate reactions can be buffered is of particular relevance.

Fourth, healthy individuals could experience social exclusion differently than individuals suffering from psychological disorders, and therefore the results may not generalize to a clinical population. Given that the present results may have particularly relevant implications for individuals suffering from specific psychological disorders, as elaborated next, replicating the present findings in clinical populations is an important step for further research.

And finally, one limitation to our results is that the effect of our intervention on hurt feelings may be accounted for by social desirability or demand effects. More specifically, participants in the OLP condition were informed about the expected effect of the OLP and thus might have answered in a way that meets the experimenters’ expectations. However, one argument against such demand effects are our findings on expected social pain reported in the Supplemental Material. In particular, following the intervention and prior to playing Cyberall, all participants were asked to indicate the extent to which they expect social pain if they were to be excluded. Demand effects to this question should manifest in lower social pain expectancies for the OLP compared to the control condition. Yet, social pain expectancies did not differ significantly between the OLP and the no-treatment condition. Descriptively, participants in the OLP condition expected even more social pain than participants in the no-treatment condition. We thus carefully conclude that participants generally might not have been prone to demand effects and our findings likely go back to the intervention resulting in less perceived social pain and hurt feelings. Nevertheless, future research may fruitfully explore ways to disentangle social desirability and demand effects from effects of the treatment alone, for instance by using more indirect measures.

The present findings add a further important piece supporting the general pain-overlap theory. At the same time, they indicate that reflexive reactions to social exclusion are susceptible to moderation, supporting the notion that the construal of social exclusion is best understood as cognitively mediated^20^. By this, our findings suggest important new practical perspectives. For instance, evidence for the effectiveness of OLPs in mitigating social pain could have practical implications for situations in which individuals are not able to rely on functional coping strategies, such as cognitive reattributions or seeking social support^57^. In such situations, OLPs could be a promising approach to reduce the immediate distress induced by social exclusion and prevent dysfunctional coping strategies (e.g., aggressive behaviors). While past research has focused on understanding such dysfunctional coping strategies^67^ but is silent about their prevalence, social exclusion is generally a frequent experience that is part of many people’s everyday life and causes strong distress^68^. Yet, conclusions from the present research are limited to self-reported affective consequences and cannot be generalized to, for instance, behavior following exclusion. Future research may thus reveal whether OLP interventions can also mitigate behavioural consequences of social exclusion such as impaired self-control and aggression^67^.

In conclusion, OLP treatments are a promising approach for treating social pain. This is noteworthy because to date, only few strategies to buffer the adverse immediate effects of social exclusion could been identified. The present findings open new avenues for helping people that frequently suffer from social pain, as it might represent a possibility to reduce maladaptive emotion regulation strategies and potentially destructive behaviors.

### Supplementary Information

Supplementary Information 1.

Supplementary Information 2.

## Data Availability

The datasets generated and/or analyzed during the current study are available in the OSF repository, https://osf.io/dxzag/.

## Code availability

The computer code used to analyze the generated data of the current study is available in the OSF repository, https://osf.io/dxzag/.
