# Supplementary material for: Effects of an open-label placebo intervention on reactions to social exclusion in healthy adults: a randomized controlled trial
Source: Sci Rep. 2023 Sep 16;13:15369. doi: 10.1038/s41598-023-42547-7 (PMC10505215; doi:10.1038/s41598-023-42547-7)
Supplement: Supplementary file 1 — Supplementary Information 1. [file 41598_2023_42547_MOESM1_ESM.pdf]

Effects of an Open-label Placebo Intervention on Reactions to Social Exclusion in Healthy Adults: A Randomized Controlled Trial

Additional Variables and Analyses

Manipulation Checks

All participants were asked to indicate their perceived closeness to the experimenter as well as their impression about the experimenter's warmth, competence, and conviction of the efficacy of OLPs (i.e., the extent to which the experimenter seemed to believe in the effect of OLPs) based on 5-point Likert scale *(1 = not at all; 5 = fully).* Since internal consistency was high ($\alpha = .74$), an overall score across all four items was calculated.

This overall score of participants' assessment of the experimenter does not correlate significantly with dependent variables need threat or hurt feelings (see Table 3 for statistics).

Table 3

Pearson's correlation and p-values of the participants' assessment of the experimenter with need threat, and social pain index

	Need Threat		Hurt Feelings	
	r	*p*	*r*	*p*
Overall rating score	-.05	.663	.04	.747
Competence	-.05	.643	-.02	.845
Conviction	-.01	.943	.12	.293
Warmth	-.05	.674	.05	.675
Closeness	-.04	.704	-.05	.692

Note. $^{*}p < .05$. $^{**}p < .01$. $^{***}p < .001$

Participants in the OLP condition were additionally asked about their personal use of painkillers in general. To identify a possibly moderating effect of the use of painkillers on the two dependent variables need threat and hurt feelings, we calculated two linear regressions models. While there was a significant main effect of inclusion status on need threat, $\beta = -4.37$, $SE = .32$, $t = -13.64$, $p < .001$, neither the main effect of use of painkillers, $\beta = -0.05$, $SE = .21$, $t = -0.19$, $p = .846$, nor the interaction between inclusion status and use of painkillers, $\beta = 0.33$, $SE = .33$, $t = -1.01$, $p = .321$, was significant. The same pattern of results occurred for the second linear regression model on hurt feelings. While there was a significant main effect of inclusion status on hurt feelings, $\beta = -3.11$, $SE = .42$, $t = -7.40$, $p < .001$, neither the main effect of use of painkillers , $\beta = 0.24$, $SE = .34$, $t = 0.72$, $p = .476$, nor the interaction was significant, $\beta = -0.05$, $SE = .43$, $t = -0.12$, $p = .906$.

Social Pain Expectancy

Participants' expectancy of social pain (i.e., the extent to which they expected social pain in case of social exclusion) correlates significantly with hurt feelings, $r = .23$, $p = .046$ and social pain, $r = .31$, $p = .007$, but not with need threat, $r = .22$, $p = .054$. Linear regression models are conducted for the two significant correlations.

The linear regression model which we conducted to investigate the interaction between social pain expectancy and social experience on hurt feelings revealed a significant main effect of social experience, $\beta = -3.86$, $SE = .35$, $t = 10.93$, $p < .001$, while the interaction effect between the two predictors social experience and expectancy of social pain, $\beta = 0.36$, $SE = .36$, $t = 1.00$, $p = .321$, was not significant.

The linear regression model which we conducted to investigate the interaction between social pain expectancy and social experience on social pain revealed a significant main effect of social experience, $\beta = -2.55$, $SE = .30$, $t = 8.51$, $p < .001$, a significant main effect of social pain expectancy, $\beta = 0.65$, $SE = .20$, $t = 3.15$, $p = .002$, and a significant

interaction effect between the two predictors social experience and social pain expectancy, β = -0.67, *SE* = .30, *t* = 2.20, *p* = .031. A simple slope analysis indicated that in the exclusion condition, higher social pain expectancy predicted higher experienced social pain, β = 0.65, *SE* = .21, *t* = 3.15, *p* = .003. This pattern was not observable in the inclusion condition, β = -0.02, *SE* = .22, *t* = -0.07, *p* = .945.

Examining social pain expectancy as a dependent variable showed that expectancy does not differ significantly between the OLP condition and the no-treatment condition, *t*(71.84) = -1.92, *p* = .059. Surprisingly, the descriptive statistics indicate, that participants in the OLP condition expected more social pain (*M* = 3.61, *SD* = 1.62) than participants in the no-treatment condition (*M* = 2.92, *SD* = 1.46).

Mood

To assess mood, all participants answered the PANAVA-KS (Schallberger, 2005), which is an adaption of the PANAS (Watson et al., 1988), the standard questionnaire for measuring dimensional description of affective states. The questionnaire consists of ten bipolar affect items (α = .91) presented on a 7-point scale, which are part of three subscales: negative activation (NA; 4 items: *"stressed – relaxed", "peaceful – upset", "calm – nervous", "worried – carefree"*; α = .88, *M* = 2.43, *SD* = 1.39), positive activation (PA; 4 items: *"full of energy – low energy", "tired – wide awake", "listless – highly motivated". "enthusiastic – bored"*; α = .85, *M* = 3.53, *SD* = 1.43) and valence (VA; 2 items: *"satisfied – dissatisfied", "unhappy – happy"*; α = .87, *M* = 4.10, *SD* = 1.67).

A 2 x 2 between subjects ANOVA[1] type III was then conducted, separately for the three dependent variables negative activation, positive activation, and valence.

Analyses revealed a significant main effect of social experience on negative activation, positive activation, and valence (see Table 4 for coefficients). Excluded participants indicate

less positive (*M* = 2.68, *SD* = 1.18) and more negative activation (*M* = 3.26, *SD* = 1.39) than included participants (*M* = 4.33, *SD* = 0.92; *M* = 1.63, *SD* = 0.81). Additionally, excluded participants (*M* = 2.74, *SD* = 1.08) indicated lower values on the valence subscale than included participants (*M* = 5.39, *SD* = 0.92). However, the interaction between social experience and treatment was not significant.

Table 4

ANOVA results for the dependent variables negative activation, positive activation, and valence as indicators for mood.

Dependent variable	Independent variable	$F(1, 70)$	η^2_p	95% CI
Negative Activation	Social Experience	36.54*	.34	[0.17, 0.48]
	Treatment	0.06	.00	[0.00, 0.03]
	Social Experience x Treatment	0.22	.00	[0.00, 0.07]
Positive Activation	Social Experience	43.85*	.39	[0.20, 0.51]
	Treatment	0.23	.00	[0.00, 0.07]
	Social Experience x Treatment	0.15	.00	[0.00, 0.07]
Valence	Social Experience	130.27*	.65	[0.50, 0.73]
	Treatment	0.34	.01	[0.00, 0.08]
	Social Experience x Treatment	1.29	.02	[0.00, 0.11]

Note. CI = confidence interval.

$^*p < .05$. $^{**}p < .01$. $^{***}p < .001$

Participants' Belief, Understanding, and Conviction

Participants in the OLP condition were asked to answer one item on their personal belief in OLPs ("*I believe open-label placebos can have an impact on social pain"; 1 = don't agree at all* to *9 = fully agree*; *M* = 6.13, *SD* = 2.02), one item on how well they understood the rationale (*1 = not at all* to *5 = fully; M* = 4.84, *SD* = 0.37) and one item if they found the rationale convincing (*1 = not at all* to *5 = fully; M* = 4.16, *SD* = 0.75).

To identify a possibly moderating effect of these three variables, we tested six linear regressions models with the predictors social experience, participant's belief, the

understanding of the rationale and the participants conviction on need threat and the social pain index (i.e., a merged score of the two variables hurt feelings and social pain). None of the six models yielded a significant interaction (see Table 5 for coefficients).

Pro Social Behavior Intention

To assess pro social behavioural intentions following social exclusion, participants were asked to indicate how interested they generally are in participating in social events organised by the centre of social psychology on a 9-point scale (*1 = not interested at all, 9 = very interested;* $M = 7.28$, $SD = 2.00$). However, there was no significant effect of social experience on the intention to behave pro socially, $t(70.92) = -1.02$, $p = .309$, as excluded ($M = 7.53$, $SD = 1.81$) compared to included ($M = 7.05$, $SD = 2.17$) individuals' intentions to participate in the event did not differ.

Table 5

Regression models of the potential moderating variables belief in the effect of OLP, understanding of the rationale and how convicted the participants were of the rationale.

DV	Moderating Variables														
	Belief					Understanding					Conviction				
	$R^2_{Adj.}$	Predictor	β	SE	T	$R^2_{Adj.}$	Predictor	β	SE	T	$R^2_{Adj.}$	Predictor	β	SE	T
Need Threat	.841	Soc. Exp.	4.43	.32	14.03***	.837	Soc. Exp.	4.43	.32	13.77 ***	.838	Soc. Exp.	4.46	-.32	13.89***
		Belief	0.21	.23	0.90		Understanding	-0.02	.27	-0.07		Conviction	-0.14	.25	-0.58
		Soc. Exp. x Belief	-0.29	.32	-0.92		Soc. Exp x Understanding	-0.00	.34	-0.01		Soc. Exp. x Conviction	0.13	.33	0.40
Social Pain Index	.606	Soc. Exp.	1.24	0.17	-5.71***	.546	Soc. Exp.	1.23	.18	6.79 ***	.614	Soc. Exp.	1.24	.18	6.80***
		Belief	0.07	.12	0.54		Understanding	0.01	.15	0.09		Conviction	-0.02	.14	-0.17
		Soc. Exp. x Belief	-0.34	.17	-2.00		Soc. Exp x Understanding	-0.09	.19	-0.48		Soc. Exp. x Conviction	-0.00	.19	-0.01

Note. DV = dependent variable. Soc. Exp. = social experience. *$p < .05$. **$p < .01$. ***$p < .001$
