# Supplementary material for: Effects of an open-label placebo intervention on reactions to social exclusion in healthy adults: a randomized controlled trial
Source: Sci Rep. 2023 Sep 16;13:15369. doi: 10.1038/s41598-023-42547-7 (PMC10505215; doi:10.1038/s41598-023-42547-7)
Supplement: Supplementary file 2 — Supplementary Information 2. [file 41598_2023_42547_MOESM2_ESM.pdf]

Effects of an Open-label Placebo Intervention on Reactions to Social Exclusion in Healthy Adults: A Randomized Controlled Trial

– Supplemental Material –

1. Rationales (Translated from German to English)

Legend: Green = placebo only; yellow = NT only; blue = important cue words; gray = actions	
**No Treatment**	**Placebo Condition**
The participant is picked up in front of the respective room. The experimenter greets the participant and the participant deposit their personal belongings by the window.	
"Hello, my name is .... I am your contact person for this study. It's great that you are taking part in our study and that you are here now!"	
Both sit down at the table. The consent form is in front of the participant. The experimenter asks the participant to read the consent form carefully and to sign it.	
"This is the consent form for the study. Please read the following sheet carefully and sign it if you agree. If you have any questions about this information, please feel free to ask me."	
Respondent asks any questions that may arise, signs and returns CF. E begins to narrate:	
"I will now explain the further procedure of the study to you. As you already know from the study information, we will randomly assign all study participants to one of the two study groups. We will shortly draw lots to determine whether you will be assigned to the open-label placebo group or the no-treatment group, i.e., the control group."<br><br>*"Before we draw the study group, I'm going to give you some information about the goal of the study and placebos. If you have any questions, feel free to interrupt me at any time."*<br><br>*"The aim of this study is to investigate the effect of open-label placebos on human information processing and emotions in a social context. Placebos look like real drugs and are deliberately packaged that way. However, placebos do not contain a pharmacologically active ingredient. It is known that placebos have a clinically significant effect, but the problem is that placebos cannot actually be used, because patients have to be deceived, as patients are led to believe that they are being given a real drug. This deception is unethical. However, it has now been discovered that placebos also work when they are administered openly, i.e., when it is known that they are placebos - this is called an open-label placebo. In this study, we now want to test this in a social context."*<br><br>*"We know from clinical research that placebos have meaningful effects on pain, depression, and anxiety. As mentioned earlier, scientists used to assume that placebo pills could only help if they were given masked – that is, with deception. As said, more recent studies indicate that placebos can work even when the patient knows it is a placebo. Many studies show that the placebo effect is very effective (strongly emphasize) for many symptoms. That is, even openly administered placebos are clinically significant and can relieve pain, cramps, and gastrointestinal distress, among other things, and also have a positive effect on mood."*<br><br>*" I will now draw a slip of paper for you that says which condition you will be assigned to."*	

<table>
<tr><td colspan="2">The experimenter draws a slip of paper and shows it to the participant. Then the experimenter enters the drawn condition into the list of participants.</td></tr>
<tr><td>"You have been assigned to the control condition, which means you will not take a placebo pill today."

"I will now explain to you how we are going to proceed in this study and what that means for you."

The control group you are in and your participation is just as important (emphasize) as the placebo group for our study. Our study is a so-called randomized controlled trial. In this kind of study - the gold standard for research – the control group – is an essential component. Only with the help of the control group can we see if the open- label placebo really has an impact on the reactions we are interested in.

As you can see, it is very important for our research to have a good control group. Accordingly, we ask you to complete the following surveys on the computer consciously and honestly."</td><td>"You have been assigned to the open-label placebo condition, which means you will take a placebo pill today."

"I will now explain to you how we are going to proceed in this study and what that means for you."

Interestingly, placebos do not only with physical symptoms, but also with so-called social pain. This social pain arises from negatively experienced social interactions (strongly emphasize). That placebos can also relieve social pain is due to the fact that physical and social pain are processed very similarly in the brain. For example, it has been shown that a placebo can reduce lovesickness in recently separated people, i.e., people who took a placebo were less lovesick than people who did not take a placebo.

But why can placebos relieve symptoms? A very important explanation is that the body can react automatically to the intake of medication. From an early age we learn that taking a pill and experiencing an effect are connected. Thus, merely swallowing the pill can relieve the pain. We also know that placebos can release neurotransmitters, such as endorphins, dopamine, or endocannabinoids, and can automatically activate certain brain areas. Neurotransmitters are chemical messengers that transmit or modulate stimuli from one nerve cell to another. These neurotransmitters can relieve pain or have a positive effect on mood."</td></tr>
<tr><td colspan="2">"As part of today's study, you will later play a short ball game on the computer. In this game, you may be in the inclusion group. That means you will get the ball the same number of times as the other players. However, it is also possible to get excluded by the other players. In this case you will get the ball less often than the others.

I don't know which group you will be assigned to because again, the assignment will be random and the computer will assign you automatically.

If you are excluded, this can lead to negative feelings and so-called social pain for a short time, but this will not go beyond the level of daily experiences.</td></tr>
</table>

	Based on the mechanisms of action of open-label placebos just mentioned, we expect that the open-label placebo will cause you to experience less social pain and fewer negative feelings should you get excluded in the ball game.<br><br>*"It's also absolutely fine if you have doubts that the placebos work. Because as mentioned earlier, placebos can work automatically, which means they can work even if you have doubts."*<br><br>*"It is important for you to know that for some people, the effect of open-label placebos sets in earlier and for other people it sets in later. It has been shown that placebos usually work very quickly, so we expect the pill to work within a few minutes. When you take the pill, we recommend that you also make yourself aware of what the placebo is supposed to do for you. I.e., that you will experience less social pain after the possible exclusion. I am aware that this may sound strange to you at first. All in all, I want to encourage you to give the open-label placebo treatment a chance today and to just watch what happens."*<br><br>*"These are the pills. P is for placebo. (Point to name). It consists only of sugar (lactose, sucrose, glucose) and stabilizers (Point to package at contains). As you can see, the pill actually contains no active ingredients. That is why we expect no side effects when taking it. Here is your pill. Feel free to pour yourself some water for it. Please swallow the pill as a whole without chewing it."*
"Do you have any other questions?"	
	Participants takes the pill.
"I will now quickly prepare something in the other room for the second part of the study, please sit here for a moment. Also, please do not use your cell phone during this time, I will be right back."	
E. goes to the lab with the participant list and enters the participant number and condition in the online survey. Then she clicks on continue and the briefing text for the participant appears on the monitor. The E. goes back to the room where the participant is waiting.	
"Now, I will take you to the other room where you will be asked to play a short game on the computer. After that, you will be asked to answer a few more questions about your experience, as well as to provide demographic information. Once you are done with the study on the computer, you can report to the experimenter in the room. Do you have any	

questions about this procedure? Once you are done with the questions, the study is over for you. Thank you for your participation!"<br><br>*Text can be said while going over to the lab*.
The E. takes the participant to his/her seat in the laboratory and returns to the room. There, the E. fills out the questionnaire intended for the E. on the iPad and prepares everything for the next participant.

2. Main Variables and Scales

4-items Need Threat Scale (Rudert & Greifeneder, 2016)

Please indicate how you felt during the Cyberball game.
During the game, I felt..

devalued	*x x x x x x x x x*	*valued*
invisible	*x x x x x x x x x*	*recognized*
rejected	*x x x x x x x x x*	*accepted*
powerless	*x x x x x x x x x*	*powerful*

Hurt feelings (Rudert & Greifeneder, 2016)

Please indicate to what extent you agree with the following statement:
The other players' behavior hurt me.

1 = completely disagree, 9 = completely agree

Social Pain

Experiencing exclusion or rejection is often accompanied by negative feelings. This is often referred to as social pain because these experiences can be very similar to physical pain.

Please rate below to what extent you are experiencing social pain at the moment.

0 = no pain, 10 = strongest pain imaginable

Manipulation Checks

The following questions are about the Cyberball game you played in this study.
To what extent did you actively participate in the ball throwing?

1 = Not at all, 9 = Very much

What percentage of all throws did you approximately receive in the Cyberball game?

__ *%*

Data Quality Checks

Please answer the following questions honestly.
There may be reasons why you did not seriously participate in this study. If this is the case, please let us know so that the quality of our data is not compromised. This will not affect your compensation. All your responses will be anonymous.

How seriously did you participate in the survey?
1 = Not at all seriously, 9 = Very seriously

Is there a reason not to use your data? If yes, what is it?

____
